# A semi‐empirical model for the therapeutic range shift estimation caused by inhomogeneities in proton beam therapy

**DOI:** 10.1120/jacmp.v13i2.3631

**Published:** 2012-03-08

**Authors:** Vadim Moskvin, Chee‐Wai Cheng, Leia Fanelli, Li Zhao, Indra J. Das

**Affiliations:** ^1^ Department of Radiation Oncology Indiana University School of Medicine Indianapolis IN 46202; ^2^ Indiana University Health Proton Therapy Center Midwest Proton Radiotherapy Institute Bloomington IN 47408‐1398 USA

**Keywords:** proton beam, range shift, high‐Z, semi‐empirical modeling, water‐equivalent thickness

## Abstract

The purpose of this study was to devise a simple semi‐empirical model to estimate the range shift in clinical practices with high‐Z inhomogeneity in proton beam. A semi‐empirical model utilizing the logarithmic dependence on Z in stopping power from Bohr's classical approach has been developed to calculate the range shift due to the presence of inhomogeneity. Range shift from metallic plates of atomic number Z of various thicknesses were measured in water using a parallel plate ionization chamber and calculated with the FLUKA Monte Carlo code. The proton range shifts for bone and polymethyl methacrylate (PMMA) were estimated using the semi‐empirical model and compared with Monte Carlo calculation. The semi‐empirical equation to determine range shift and water equivalent thickness is presented. The model predicts a shift of the therapeutic range to within 2.5% accuracy for initial proton energies of 50 to 250 MeV and atomic numbers from 3.3 (effective Z for water) to 82. This equation is independent of beam energy, and thus provides range shift from high‐Z materials without the knowledge of proton energy. The proposed method of calculating the therapeutic range shift accurately requires only knowledge of the effective or actual atomic number of the inhomogeneity and the thickness of the inhomogeneity along the beam direction. The model generalizes the range shift calculation for any material based on its effective atomic number, and permits reliable prediction of the range shift for material combinations where no data is currently available. The proposed model can be readily implemented in routine clinical practice for proton range shift estimation and quality assurance on the treatment planning.

PACS numbers: 87.53.‐j, 87.55.‐x, 87.53.Bn, 87.55.D‐, 87.55.Qr, 87.55.K‐

## I. INTRODUCTION

The most important advantage of heavy charged particles in radiation therapy is that they deposit much of their energy within a finite narrow range represented by the Bragg peak.^(1)^ The dose deposition behavior is utilized to deliver dose to tumor with high level of accuracy and to spare normal tissues distally. A large number of patients who undergo proton treatments may have inhomogeneities in their bodies, such as dental fillings, prosthetic devices, metallic reconstruction, and fiducial markers. These inhomogeneities produce perturbation in dose deposition and range shift in particle beam therapy.

The effect of high‐Z materials in proton therapy has been studied by various investigators. Urie et al.^(2)^ presented a qualitative approach and documented the presence of dose perturbation caused by realistic inhomogeneities. Recently, Herrmann et al.^(3)^ and Cheung et al.^(4)^ investigated the effect of Ni‐Ti and ceramic carbon‐coated fiducial markers on charged particle therapy. The significant dose perturbation caused by such inhomogeneities has raised concerns over the usage of such fiducial markers close to the particle range.^(5)^

Impact on proton transport due to the thickness of an inhomogeneity is commonly estimated by its water equivalent thickness (WET)^(6)^ or water equivalent distance (WED).^(7)^ Various approaches have been reported to predict the WET in particle beams.^(^ ^8^ ^–^ ^12^ ^)^ A method to compensate for the inhomogeneities has also been suggested.^(2)^ However, Gottschalk^(8)^ commented on and cautioned concerning the use of analytical method proposed by Zhang and Newhauser^(11)^ due to differences in mean excitation energy that could produce error in the computation of ranges. A detailed description of the excitation energy and computation of range has also been proposed by Bichsel.^(13)^ Nichiporov et al.^(5)^ recently presented experimental measurements of the range shift for a number of inhomogeneities at the depths corresponding to the middle of a spread out Bragg peak (SOBP). The usage of these methods for WET calculations requires a significant amount of tabulated data from the PSTAR stopping power database or the ICRU Report 49,^(^ ^14^ ^,^ ^15^ ^)^ as well as a knowledge of the initial energy of proton beams.

Clinical practice typically operates using the concept of the required (modulated) proton range, instead of using the actual energy of the proton beam. Therefore, in order to be useful in a routine clinical practice, the technique to calculate range shift should not require knowledge of the proton beam energy. For this purpose, a semi‐empirical model for the estimation of the proton range shift in the presence of inhomogeneity is developed in this study.

## II. MATERIALS AND METHODS

### A. A model for range shift calculations

**Figure 1:**
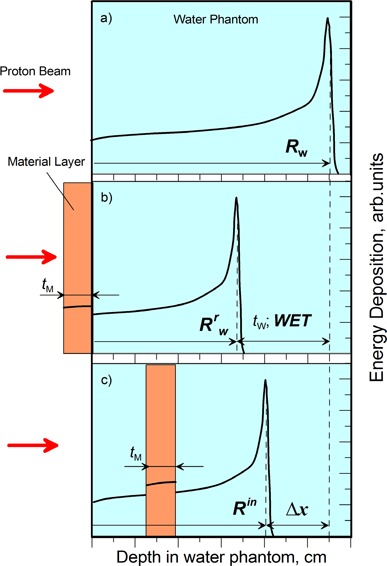
Geometry used to define: (a) the range of the protons in water phantom $R_{w}$; (b) water equivalent thickness WET, range shift in the presence of inhomogeneity on the surface of water phantom (typical experimental setup to determine WET); and (c) observed range shift Δx in the case of the inhomogeneity inside the water phantom.

We start by assuming the geometry in Fig. 1, where Fig. 1(a) represents the depth‐dose characteristics of proton beam in a homogenous water phantom where $R_{w}$ represents the range (90%) in water. Consider a slab of thickness $t_{M}$ (in cm) of a material, M, placed in the front of a water tank (Fig. 1 (b)). A unidirectional proton beam with energy in the range 50 to 250 MeV is incident normally to the surface M. The amount of energy deposited in the slab corresponds to the amount of energy deposited in a slab of water equivalent thickness (WET), $t_{W}$, is defined as:

(1) $$WET(t_{M},Z)=t_{M}\frac{\rho_{M}}{\rho_{W}}{\left(\frac{S}{\rho }\right)}_{W}^{M}$$

where $\rho_{M}$ is the mass density of the material, $\rho_{W}$ is the mass density of water, *Z* is the atomic number of the material *M*, and ${\left(\frac{S}{\rho }\right)}_{W}^{M}$ is the mass stopping power ratio of material *M* to water *W*. Implicitly, the mass stopping ratio depends on *Z* and *E*.

Equation (1) for the water equivalent thickness corresponds to the definition given by Zhang et al.^(^ ^11^ ^,^ ^12^ ^)^ Figure 1(b) represents the geometry used in WET definition.

A clinical example of the geometry in Fig. 1(b) is the placement of fiducial markers on the patient's surface. A more commonly encountered situation is that of an inhomogeneity inside a patient, such as surgical clips, spinal prosthesis, metallic breast implants, dental fillings, and hip prosthesis, as illustrated by the schematic diagram in Fig. 1(c).

The proton range $R_{\text{in}}$ in Fig. 1(c) is defined as the range of protons in an inhomogeneous system consisting of water embedded with a layer of material M. Medin and Andreo^(16)^ showed that the mass stopping power ratio of air to water, ${\left(\frac{S}{\rho }\right)}_{air}^{W}$, changes slowly as a function of depth up to the proximal slope of the Bragg peak in a homogeneous medium where the Bragg peak starts forming. The analysis of the ICRU Report 49 data^(^ ^14^ ^,^ ^15^ ^)^ shows that the mass stopping power ratio of a material to water has a weak dependence on proton energy above 50 MeV for low‐Z (for example, about 2% for Z=13) and above 80 MeV for high‐Z (about 5% for Z=82) materials. Thus, in our semi‐empirical model, the slab M is placed before the Bragg peak in water for a given energy, and the thickness of the slab $t_{M}$ satisfies the condition $t_{M}<\text{RM}.$

The observed range shift, Δx, can be defined as $R_{W}-R_{W}^{r}-t_{M}$, where $R_{W}$ is the range of protons in water, and $R_{W}^{r}$ is the reduced range of protons in water due to the presence of material M (see Fig. 1). The difference, $R_{W}-R_{W}^{r}$, is equal to the water equivalent thickness, WET, of the material (Eq. (1)). The observed proton's range shift, and consequently the therapeutic range shift $R_{90}$, in a water phantom embedded with a layer $t_{M}$ of a material M with atomic number Z is then given by:

(2) $$\Delta x(t_{M},Z)=WET(t_{M},Z)-t_{M}$$

The function, $\text{WET}(t_{M},Z)$ has a monotonic dependence on atomic number Z that was fitted by a function as shown in Eq. (3a):

(3a) $$WET(t_{M},Z)=t_{M}\frac{\rho_{M}}{\rho_{W}}\alpha (Z)$$

Where α(Z) is the fitting function expressing the dependence of ${\left(\frac{S}{\rho }\right)}_{W}^{M}$ on atomic number Z of the material.

For a particular case where $\rho_{W}=1\;g/{\text{cm}}^{2}$, Eq. 3(a) can be simplified further as:

(3b) $$WET(t_{M},Z)=t_{M}\rho_{M}\alpha (Z)\;\;\;\;\;\;[\text{cm}]$$

Final equations in the Results and Discussion section below will also be written in a simple form omitting the $\rho_{W}$.

### B. A fitting procedure

According to Bohr's classical approach for a universal form of the stopping power for ions, the stopping number $L_{\text{Bohr}}$ expresses logarithmic functional dependence on target atomic number.^(17)^ A semi‐empirical model with a logarithmic dependence on atomic number was used to determine the stopping powers for ions with energies of 0.1–1.0MeV/u in elemental targets.^(18)^ In the present work, we used the functional form A−B*Ln(Z) to represent α(Z).

The tabulated stopping power data from NIST^(15)^ were used for fitting α(Z), with a least squares fitting routine from the Golden Software Grapher (v. 7).

### C. Generalization of semi‐empirical model

Equation (3) generalizes range shift for any material based on the effective atomic number. The calculations presented in Results and Discussion below uses the effective atomic number, $Z_{\text{eff}}$, for protons. Please note that $Z_{\text{eff}}$ depends on the type of radiation and interaction probability.

A number of studies have discussed the definition of effective atomic number^(^ ^19^ ^–^ ^26^ ^)^ for photons and protons over a wide range of energies. The conventional method to determine $Z_{\text{eff}}$ of a material for photon transport is based on the photoelectric coefficient per electron or total photon energy absorption cross section per electron in composition,^(^ ^19^ ^,^ ^22^ ^)^ or total photon interaction cross section per atom^(^ ^21^ ^,^ ^23^ ^,^ ^24^ ^)^ for each element in the material. The estimated value of the $Z_{\text{eff}}$ varies with the photon energy due to the weighting of the photoelectric interaction process and depends on the calculation technique used. The weighted average of the number of electrons per atom gives an estimation of the average atomic number of a material consisting of multiple elements.^(22)^

On the other hand, charged particles undergo multiple interactions during their transport through an absorbing medium. Collisions with the atomic electrons dominate in the region of the therapeutic energies. The procedure of the $Z_{\text{eff}}$ calculation for charged particles involves the utilization of the total stopping power.^(^ ^20^ ^,^ ^24^ ^)^ The values of the $Z_{\text{eff}}$ thus calculated are different from those obtained for the conventional megavoltage photons. For instance, the classical value for $Z_{\text{eff}}$ for water for photons given by Johns and Cunningham^(19)^ is 7.4,^(^ ^19^ ^,^ ^22^ ^)^ while it is 3.3 for protons as determined Prasad et al.^(20)^ The values for $Z_{\text{eff}}$ calculated by Prasad et al. is used in the Results and Discussion section.

### D. Validation of the semi‐empirical model

The experimental setup used to validate the model corresponds to the geometry presented in Fig. 1(b). The depth‐dose data were collected for the various high‐Z materials: Aluminum (Al), Titanium (Ti), Copper (Cu), Tin (Sn), and Lead (Pb) using a parallel plate ion chamber. Slabs of different high‐Z materials of various thicknesses were attached to the upstream face of the water tank during the depth‐dose scans. A set of measurements was taken to measure WET and range shift.

The values of the therapeutic range $R_{90}$ (defined as the 90% depth in water for a given proton energy) were determined from the depth‐dose scans. The water equivalent thickness $\text{WET}(t_{M},Z)$ was derived from these data. The measured data on $\text{WET}(t_{M},Z)$ was compared to the results obtained by Eq. (3) with the use of its final form that is given by Eq. (4) in the Results and Discussion section.

The results of range shift from measurements and from the semi‐empirical model were compared with the results of simulation using the general‐purpose particle transport code FLUKA^(^ ^27^ ^,^ ^28^ ^)^ version 2008.3c.0. The capability of FLUKA for proton therapy calculations has been validated in a number of studies.^(^ ^29^ ^–^ ^32^ ^)^ The model of the beam used in simulation of the range shift experiments with pure materials was verified by independent measurements on depth‐dose distribution in a water equivalent phantom.

One of the FLUKA defaults, PRECISIO, was used to customize the physical model used in the simulation. The initial proton transport was simulated with a cutoff energy at 100 keV. USRBIN cards were used for scoring the particle fluence and dose with the parameter DOSE. The geometry of the irradiated phantom was defined with combinatory geometry package, which is a part of the FLUKA code. Fifty million $\left(5*10^{7}\right)$ initial protons were used in the simulation.

The range shift calculated by semi‐empirical model is compared with the Monte Carlo simulation for bone (atomic composition in percentage: H (6.3984); C (27.8); N (2.7), O (41.0016), Mg (0.2), P (7.0), S (0.2), Ca (14.7); mass density $1.85\;\text{gm}/{\text{cm}}^{3},\;Z_{\text{eff}}=4.9$,^(20)^ and polymethyl methacrylate (PMMA) (atomic composition in percentage: H (8.064); C (60.055); O (31.999); mass density 1.19 g/cm^3^, Zeff=3.3.^(20)^ The bone composition was based on the ICRU compact bone data taken from the standard FLUKA database of the materials.

**Figure 2:**
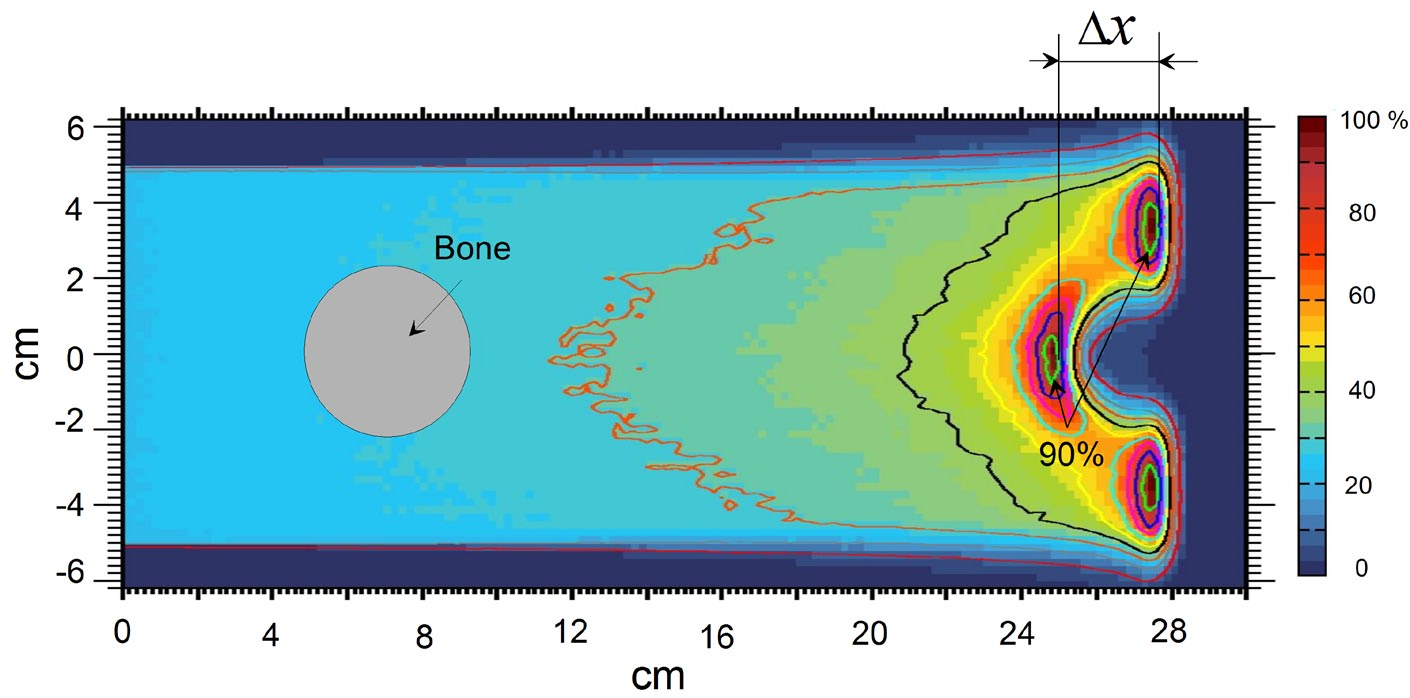
Range shift in the presence of the bone material in a water phantom. Monte Carlo simulated dose in the water phantom in the presence of the 4 cm diameter cylinder, imitating the bone. Isodose line corresponding to 90% dose defines the range of protons along the normal to the surface of the beam incidence.

An example of model application in complex geometry is shown in the Appendix utilizing the Monte Carlo simulation for range shift estimation. The geometry in Fig. 2 contains a bone embedded inside a water equivalent phantom. The bone was represented by a cylinder (4 cm in diameter). A mono‐energetic proton beam of energy 208.4 MeV and of $10\times 10\;{\text{cm}}^{2}$ field size impinged normally on the surface of the phantom. The range shift is estimated as the shift of the 90% isodose line.

## III. RESULTS & DISCUSSION

### A. A semi‐empirical equation for range shift

**Figure 3:**
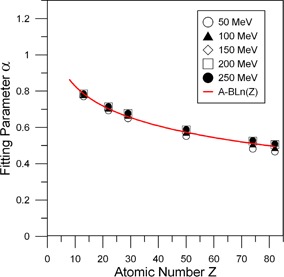
Dependence of the fitting function α(Z), representing the ratio ${\left(\frac{S}{\rho }\right)}_{W}^{M}$ of mass stopping power in material to water for various initial energies of protons. The symbols are the calculated values using the tabulated data on mass stopping power.^(14)^ The curve is the fitted function for α(Z).

The function α(Z) calculated using tabulated stopping power data^(15)^ is plotted in Fig. 3 (symbols) for various initial energies of proton beams. Since the dependence on energy is weak at E>50MeV for low‐Z and E>80MeV for high‐Z, a single set of fitted parameters may be used over the energy interval up to 250 MeV of the initial proton energies. The fitting parameters A and B in the functional form A−B*Ln(Z) were determined to be 1.192 and 0.158, respectively. The fitting function is presented as a solid line in Fig. 3.

A residual value is defined as the difference between the fitted value and the actual measured data values for a given Z or E value. The residual sum of squares (SSE) is the sum of the squares of all the residual values and is a measure of the quality of the fit in a least squares fitting method. The SSEs over the atomic number range from water to lead are 0.00265, 0.000205, 0.000337, 0.000839, and 0.00136 for initial proton energies of 50, 100, 150, 200, and 250 MeV, respectively. On the other hand, the variation of SSE, cumulative over the energy range from 50 to 250 MeV, with the atomic number is from $5.6*10^{-5}$ for water to 0.0012 for lead suggesting that the model is extremely accurate.

Using Eq. (3), the final form of the empirical equation for calculation for the water equivalent thickness $\text{WET}(t_{M},Z)$ is given as:

(4) $$WET(t_{M},Z)=t_{M}\rho_{M}(1.192-0.158\text{Ln}(Z))\;\;\;\;\;[\text{cm}]$$

The observed range shift Δx in the presence of an inhomogeneity in an irradiated volume is then calculated according to Eq. (2) and (4) as:

(5) $$\Delta x(t_{M},Z)=t_{M}[\rho_{M}(1.192-0.158\text{Ln}(Z))-1]\;\;\;\;[\text{cm}]$$

### B. Accuracy of the semi‐empirical model

**Figure 4:**
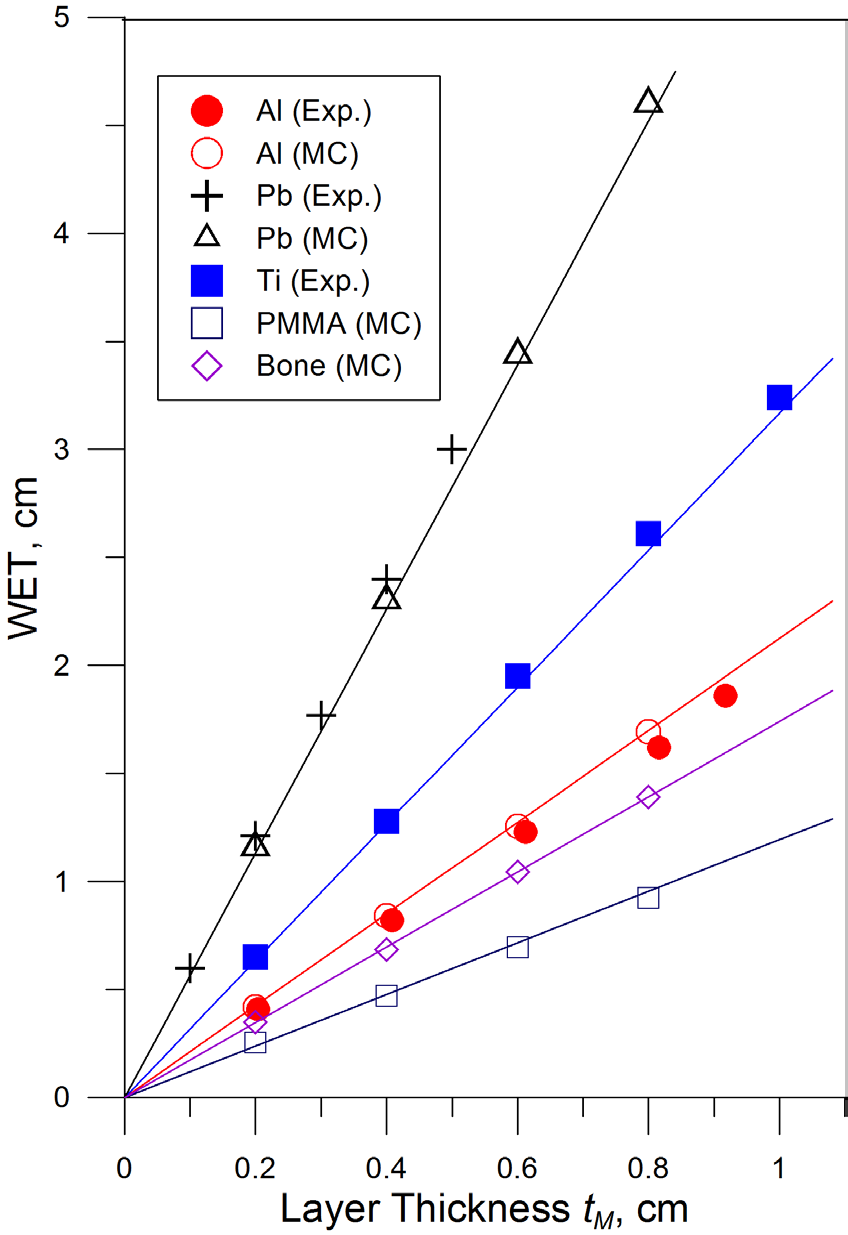
Comparison of the measurements (filled symbols), Monte Carlo simulations (open symbols) and results predicted by the model as described by Eq. (4) (line). Physical densities of Al, Ti, Cu, Pb used in the calculations are 2.7, 4.5, 8.96, and 11.4 g/cm^3^, respectively. Published values for polymethyl methacrylate (PMMA) (mass density 1.19 g/cm^3^, effective atomic number $Z_{\text{eff}}=3.3$ ^(19)^) and bone (mass density $1.85\;\text{gm}/{\text{cm}}^{3},\;Z_{\text{eff}}=4.9$ ^(19)^) are used. Proton beam is defined by range of $R_{90}=15.84\;\text{cm}$ in water.

Figure 4 illustrates a comparison of the measured data on $\text{WET}(t_{M},Z)$ with Eq. (3) for a proton range $R_{90}=15.84\;\text{cm}$ in water for various materials. It can be seen from Fig. 4 that the measured data is well represented by Eq. (4). The deviations of the predicted values calculated from Eq. (4) are within 1.5% from the measurements for the ranges of proton beams used.

The $\text{WET}(t_{M},Z)$ data calculated with the Monte Carlo simulation for Al, Pb, PMMA, and bone shown as open symbols in Fig 4. It is seen from the figure that Monte Carlo simulated data are close to the results given by Eq. (4) for Pb and Al and those given by the measurements. Equation (4) also accurately predicts the WET values calculated with the Monte Carlo simulation for the materials with the atomic number different from pure materials, like for PMMA and bone given in Fig. 4.

**Figure 5:**
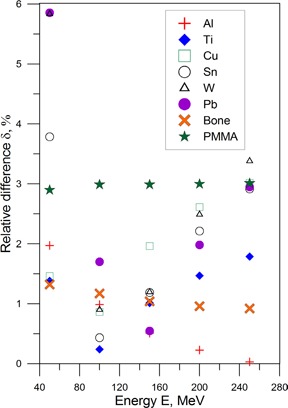
Relative percent difference δ between the semi‐empirical fitting by Eq. (4) and calculations using the data from PSTAR^(14)^ in Eq. (1).

To examine the effect of heterogeneities on $\text{WET}(t_{M},Z)$ and the corresponding range shift, the values obtained from Eq. (4) are compared with those from Eq. (1) using the tabulated data on mass stopping power.^(15)^ The accuracy of the analytical model is shown in Fig. 5 where the relative percent difference δ between two calculations of WET is presented. For the initial proton energy of 100 MeV, the difference between the two equations is about 1% for low‐ and medium‐Z values (Al, Ti, Cu, Sn). It is about 1% for Tungsten (W), and 1.8% for Lead (Pb). The value of δ increases with energy to 2.2% for 180 MeV, and reaches 2.5% at 200 MeV. However δ is still significantly below 1% for low‐Z materials (Al) at 200 MeV. The WET values between the two equations in the low‐energy region from 50 to 100 MeV agree to about 2% for low‐Z materials and about 2.3 % for high‐Z materials from 70 MeV to 100 MeV. Considering the compounds, the WET values for bone agrees with an accuracy of ~1% for all energies; however, PMMA has an error of about 3% in the considered interval of energies from 50 MeV to 250 MeV. Generally, the fitting parameters predict the WET and, consequently, the shift of the therapeutic range $R_{90}$ and 90% depth to within ±3% accuracy for initial proton energies of 50 to 200 MeV, and atomic numbers from 3.3 (effective atomic number $Z_{\text{eff}}$ for water^(20)^) to 82.

## IV. CONCLUSIONS

A semi‐empirical model for range shift as shown in Eqs. (4) and (5) provides a simple method to accurately estimate the range shift. The proposed method requires only knowledge on the effective or actual atomic number of the inhomogeneity, the physical density, and the thickness of the inhomogeneity along the beam direction. The results obtained from the semi‐empirical model are in good agreement with measurements and Monte Carlo simulation.

The model generalizes the range shift calculation on any material based on its effective atomic number, not just the materials listed in ICRU Report 49, and permits reliable prediction of the range shift for material combinations where no data are currently available. The proposed model can be readily implemented in routine clinical practice in order to check the accuracy of a treatment plan.

## ACKNOWLEDGMENTS

The authors wish to thank Dr. D. Nichiporov for useful discussions. The authors are also grateful to reviewers of the manuscript for their valuable comments and suggestions.

## Appendix

The application of the semi‐empirical model for calculations in a complex geometry is illustrated by the example of range shift estimation for bone material in water phantom. Figure 2 presents a color wash dose distribution from Monte Carlo simulation of proton transport through the bone equivalent material as described in Section D of Materials & Methods above. A shift of the Bragg peak and the corresponding 90% isodose line due to presence of the bone is clearly demonstrated. The estimated $R_{90}$ range shift, Δx, is about 3 cm upstream along the beam axis.

On the other hand, applying Eq. (5) with $Z_{\text{eff}}=4.9$,^(19)^ our semi‐empirical model predicts a range shift Δx of 2.96 cm along the beam axis passing through the center of the bone, in good agreement with that from Monte Carlo simulation.
